# Crystal structure of butane-1,4-diyl bis­(furan-2-carboxyl­ate)

**DOI:** 10.1107/S2056989019007175

**Published:** 2019-05-24

**Authors:** Mitsutoshi Hoshide, Hyuma Masu, Yuji Sasanuma

**Affiliations:** a Department of Applied Chemistry and Biotechnology, Graduate School and Faculty of Engineering, Chiba University, 1-33 Yayoi-cho, Inage-ku, Chiba 263-8522, Japan; b The Center for Analytical Instrumentation, Chiba University, 1-33 Yayoi-cho, Inage-ku, Chiba 263-8522, Japan

**Keywords:** crystal structure, model compound of poly(butyl­ene 2,5-furandi­carboxyl­ate), all-*trans* structure, C—H⋯O hydrogen bond, C—H⋯π inter­action

## Abstract

The asymmetric unit of the title compound, C_14_H_14_O_6_, a monomeric compound of poly(butyl­ene 2,5-furandi­carboxyl­ate), consists of one half-mol­ecule, the whole all-*trans* mol­ecule being generated by an inversion centre. In the crystal, the mol­ecules are inter­connected *via* C—H⋯O inter­actions, forming a mol­ecular sheet parallel to (10). The mol­ecular sheets are further linked by C—H⋯π inter­actions.

## Abstract 2

The asymmetric unit of the title compound consists of one half-mol­ecule, the whole all-*trans* mol­ecule being generated by an inversion centre. In the crystal, the mol­ecules inter­connected by C—H⋯O and C—H⋯π inter­actions.

## Chemical context   

To suppress global warming, materials derived from fossil fuels have been attempted to be replaced with plant-based products. For example, plant-derived furan-2,5-di­carb­oxy­lic acid is expected to be substituted for terephthalic acid, raw materials of aromatic polyesters such as poly(ethyl­ene terephthalate) and poly(butyl­ene terephthalate) (abbreviated herein as PBT) (Gandini *et al.*, 2016 ▸); therefore, in the future, the substitute for PBT will possibly be poly(butyl­ene 2,5-furandi­carboxyl­ate) (PBF), the alternate copolymer of furan-2,5-di­carb­oxy­lic acid and butane-1,4-diol.

The ultimate mechanical stiffness of polymers mostly corresponds to the crystalline modulus in the chain-axis direction at 0 K and depends largely on the chain conformation (Kurita *et al.*, 2018 ▸). Therefore, it is of significance to determine conformations of polymers in crystal and to relate such structural information to their mechanical properties. PBT is known to exhibit two crystal structures of α and β forms (Yokouchi *et al.*, 1976 ▸; Desborough & Hall, 1977 ▸). The α form adopts *gauche* ^+^ (*g* ^+^), *gauche* ^+^ (*g* ^+^), *trans* (*t*), *gauche* ^−^ (*g* ^−^) and *gauche* ^−^ (*g* ^−^) conformations in the O—CH_2_—CH_2_—CH_2_—CH_2_—O unit (referred hereafter to as the spacer), while the β form has a near all-*trans* spacer. It is known that mechanical stresses induce the α-to-β transformation, which will absorb impact and avoid fracture. Owing to such remarkable structural characteristics, PBT has been used for engineering plastics superior in impact resistance.

Single crystal X-ray structure analysis of butane-1,4-diyl dibenzoate (BT), a model compound of PBT, showed that its spacer lies in a *tgttt* conformation different from that of PBT (Palmer *et al.*, 1985 ▸). A powder X-ray diffraction study on PBF (Zhu *et al.*, 2013 ▸) has estimated dihedral angles of its spacer to be 180° (*trans*), 66° (+*synclinal*), 99° (+*anti­clinal*), 124° (+*anti­clinal*) and 148° (+*anti­clinal*) and hence quite different from those of PBT and BT. In this study, we have conducted a single-crystal X-ray diffraction experiment on a model compound of PBF, butane-1,4-diyl bis­(furan-2-carboxyl­ate) (BF), to investigate its spacer conformation and inter­molecular inter­actions and compare them with those of PBF, BT and PBT.
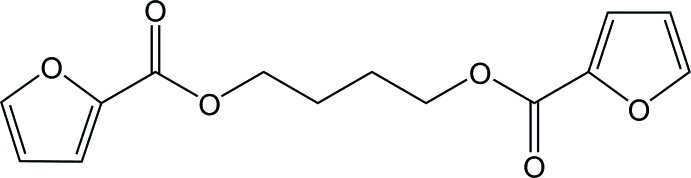


## Structural commentary   

**Figure 1:**
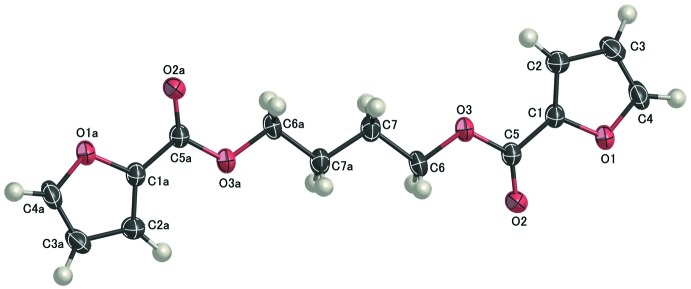
The mol­ecular structure of the title compound, showing the atom-labelling scheme. Atoms with suffix a are generated by the symmetry operation (−*x* + 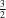, −*y* + 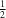, −*z*). Displacement ellipsoids are drawn at the 50% probability level. H atoms are represented by spheres of arbitrary size.

The BF spacer of the title compound adopts an all-*trans* conformation (Fig. 1 ▸), which is different from those of PBF as well as PBT and BT. The unit cell includes four mol­ecules, each of which is located on an inversion centre, and hence one half-mol­ecule corresponds to the asymmetric unit. The furan O1/C1–C4 ring is planar, while the carb­oxy O2/C5/O3 plane is slightly twisted form the furan ring, with a dihedral angle of 4.00 (15)°.

## Supra­molecular features   

**Figure 2:**
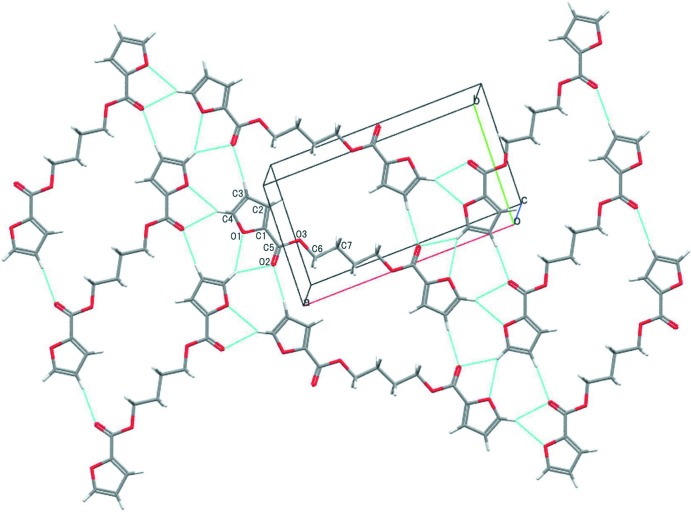
A packing diagram of the title compound, showing the mol­ecular sheet formed by C—H⋯O inter­actions (blue lines).

**Figure 3:**
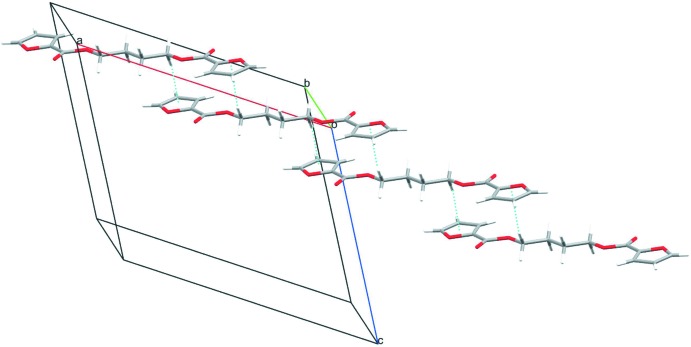
A packing diagram of the title compound, showing the inter­molecular C—H⋯π inter­actions (blue dotted lines) between the mol­ecular sheets.

In the crystal, the BF mol­ecules are inter­connected by C—H⋯O inter­actions (Table 1 ▸) to form a mol­ecular sheet parallel to (10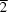) (Fig. 2 ▸). The sheets are further linked *via* a C—H⋯π inter­action (Table 1 ▸ and Fig. 3 ▸), forming a three-dimensional network. In the BT crystal (Palmer *et al.*, 1985 ▸), the benzene rings face to each other to form inter­molecular π–π inter­actions with centroid–centroid distances of 4.169 (2) and 3.910 (2) Å. In addition, the benzene rings act as donors in C—H⋯π inter­actions. As stated above, BF seems to prefer the C—H⋯O inter­actions and adopt a spacer conformation so as to fulfill the C—H⋯O inter­actions efficiently, whereas BT and PBT (Yokouchi *et al.*, 1976 ▸; Desborough & Hall, 1977 ▸) tend to adapt a spacer conformation to form π–π inter­actions.

## Database survey   

A search in the Cambridge Structural Database (Version 5.40, last update February 2019; Groom *et al.*, 2016 ▸) for BF itself gave only one similar compound, PBF (Zhu *et al.*, 2013 ▸), mentioned above. Although a search for dimethyl furan-2,5-di­carboxyl­ate (DMF-2,5-DC) gave no hits, 20 compounds related to furan-2,5-di­carb­oxy­lic acid (FDCA) were suggested as similar compounds. They are FDCA itself (Martuscelli & Pedone, 1968 ▸) and complexes including FDCA. The crystal structure of dimethyl furan-2,4-di­carboxyl­ate (DMF-2,4-DC) was reported (Thiyagarajan *et al.*, 2013 ▸). DMF-2,4-DC forms π–π inter­actions between the furan rings with centroid–centroid distances of 3.6995 (12) and 3.7684 (14) Å, and C—H⋯O inter­actions [C⋯O = 3.333 (2), 3.276 (3) and 3.465 (2) Å]. The dihedral angles between the carb­oxy group and the furan ring are 1.11–5.86°.

## Synthesis and crystallization   

Furan-2-carbonyl chloride (2.2 ml, 22 mmol) was added dropwise under a nitro­gen atmosphere to butane-1,4-diol (0.89 ml, 10 mmol) and pyridine (6.0 ml) put in a three-necked flask dipped in ice–water and stirred at room temperature for 28 h. The reaction mixture was extracted with chloro­form (10 ml) and water (10 ml), and the organic layer was washed thrice with aqueous sodium bicarbonate (10%), dried over anhydrous sodium sulfate overnight and filtrated. The filtrate was condensed on a rotary evaporator, and the residue was dried *in vacuo* and identified by ^1^H and ^13^C NMR as BF (yield 73%).

A small amount of BF was dissolved in benzene in a small phial, which was put in a larger phial including a small volume of *n*-hexane. The outer vessel was capped and stood still. After a few weeks, single crystals were found to precipitate at the bottom of the inner phial.

## Refinement   

Crystal data, data collection and structure refinement details are summarized in Table 2 ▸. All H atoms were geometrically positioned with C—H = 0.95 and 0.99 Å for the aromatic and methyl­ene groups, respectively, and were refined as riding with *U*_iso_(H) = 1.2*U*_eq_(C).

## Supplementary Material

Crystal structure: contains datablock(s) I. DOI: 10.1107/S2056989019007175/is5514sup1.cif

Structure factors: contains datablock(s) I. DOI: 10.1107/S2056989019007175/is5514Isup3.hkl

CCDC reference: 1916720

Additional supporting information:  crystallographic information; 3D view; checkCIF report

## Figures and Tables

**Table 1:** Hydrogen-bond geometry (Å, °) *Cg*1 is the centroid of the O1/C1–C4 ring.

*D*—H⋯*A*	*D*—H	H⋯*A*	*D*⋯*A*	*D*—H⋯*A*
C3—H3⋯O2^i^	0.95	2.41	3.3526 (15)	174
C4—H4⋯O1^ii^	0.95	2.60	3.4142 (18)	145
C4—H4⋯O2^ii^	0.95	2.49	3.317 (2)	146
C6—H6*B*⋯*Cg*1^iii^	0.99	2.66	3.5869 (16)	156

Symmetry codes: (i) 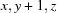; (ii) 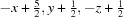; (iii) 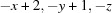.

**Table 2:** Experimental details

Crystal data	
Chemical formula	C_14_H_14_O_6_
*M*_r_	278.25
Crystal system, space group	Monoclinic, *C*2/*c*
Temperature (K)	173
*a*, *b*, *c* (Å)	16.1298 (17), 7.8773 (8), 13.5247 (14)
β (°)	123.6698 (12)
*V* (Å^3^)	1430.2 (3)
*Z*	4
Radiation type	Mo *K*α
μ (mm^−1^)	0.10
Crystal size (mm)	0.40 × 0.20 × 0.20

Data collection	
Diffractometer	Bruker APEXII CCD area detector
Absorption correction	Multi-scan (*SADABS*; Sheldrick, 1996 ▸)
*T*_min_, *T*_max_	0.94, 0.98
No. of measured, independent and observed [*I* > 2σ(*I*)] reflections	3968, 1625, 1254
*R*_int_	0.038
(sin θ/λ)_max_ (Å^−1^)	0.650

Refinement	
*R*[*F* ^2^ > 2σ(*F* ^2^)], *wR*(*F* ^2^), *S*	0.041, 0.098, 1.03
No. of reflections	1625
No. of parameters	91
H-atom treatment	H-atom parameters constrained
Δρ_max_, Δρ_min_ (e Å^−3^)	0.40, −0.25

Computer programs: *APEX2* and *SAINT* (Bruker, 2013 ▸), *SHELXS97* (Sheldrick, 2008 ▸), *SHELXL2013* (Sheldrick, 2015 ▸), *XSHEL* (Bruker, 2013 ▸), *PLATON* (Spek, 2009 ▸) and *XCIF* (Bruker, 2013 ▸).

## supplementary crystallographic information

### Crystal data

**Table:**

C_14_H_14_O_6_	*F*(000) = 584
*M**_r_* = 278.25	*D*_x_ = 1.292 Mg m^−^^3^
Monoclinic, *C*2/*c*	Mo *K*α radiation, λ = 0.71073 Å
*a* = 16.1298 (17) Å	Cell parameters from 1288 reflections
*b* = 7.8773 (8) Å	θ = 3.0–26.3°
*c* = 13.5247 (14) Å	µ = 0.10 mm^−^^1^
β = 123.6698 (12)°	*T* = 173 K
*V* = 1430.2 (3) Å^3^	Prismatic, colourless
*Z* = 4	0.40 × 0.20 × 0.20 mm

### Data collection

**Table:**

Bruker APEXII CCD area detector diffractometer	1625 independent reflections
Radiation source: fine-focus sealed tube	1254 reflections with *I* > 2σ(*I*)
Graphite monochromator	*R*_int_ = 0.038
Detector resolution: 8.3333 pixels mm^-1^	θ_max_ = 27.5°, θ_min_ = 3.0°
φ and ω scans	*h* = −20→19
Absorption correction: multi-scan (SADABS; Sheldrick, 1996)	*k* = −9→10
*T*_min_ = 0.94, *T*_max_ = 0.98	*l* = −17→16
3968 measured reflections	

### Refinement

**Table:**

Refinement on *F*^2^	0 restraints
Least-squares matrix: full	Hydrogen site location: inferred from neighbouring sites
*R*[*F*^2^ > 2σ(*F*^2^)] = 0.041	H-atom parameters constrained
*wR*(*F*^2^) = 0.098	*w* = 1/[σ^2^(*F*_o_^2^) + (0.0485*P*)^2^ + 0.4241*P*] where *P* = (*F*_o_^2^ + 2*F*_c_^2^)/3
*S* = 1.03	(Δ/σ)_max_ = 0.001
1625 reflections	Δρ_max_ = 0.40 e Å^−^^3^
91 parameters	Δρ_min_ = −0.25 e Å^−^^3^

### Special details

**Table:**

Experimental. SADABS (Sheldrick, 1996)
Geometry. All esds (except the esd in the dihedral angle between two l.s. planes) are estimated using the full covariance matrix. The cell esds are taken into account individually in the estimation of esds in distances, angles and torsion angles; correlations between esds in cell parameters are only used when they are defined by crystal symmetry. An approximate (isotropic) treatment of cell esds is used for estimating esds involving l.s. planes.

### Fractional atomic coordinates and isotropic or equivalent isotropic displacement parameters (Å^2^)

**Table:**

	*x*	*y*	*z*	*U*_iso_*/*U*_eq_	
C1	1.06223 (8)	0.66487 (14)	0.16074 (10)	0.0256 (3)	
C2	1.01643 (9)	0.81730 (15)	0.13072 (12)	0.0327 (3)	
H2	0.9468	0.8379	0.0893	0.039*	
C3	1.09319 (10)	0.94116 (16)	0.17360 (12)	0.0374 (3)	
H3	1.085	1.0609	0.1664	0.045*	
C4	1.17928 (10)	0.85557 (16)	0.22604 (12)	0.0362 (3)	
H4	1.243	0.907	0.2627	0.043*	
C5	1.02744 (8)	0.48957 (14)	0.14292 (10)	0.0246 (3)	
C6	0.88565 (9)	0.31465 (14)	0.06770 (11)	0.0287 (3)	
H6A	0.9159	0.2497	0.1427	0.034*	
H6B	0.8977	0.2524	0.0132	0.034*	
C7	0.77555 (9)	0.33626 (15)	0.01192 (12)	0.0318 (3)	
H7A	0.7459	0.3994	−0.0637	0.038*	
H7B	0.7646	0.4033	0.0656	0.038*	
O1	1.16329 (6)	0.68497 (10)	0.21987 (8)	0.0318 (2)	
O2	1.08020 (6)	0.36607 (10)	0.17085 (8)	0.0341 (2)	
O3	0.92865 (6)	0.48359 (10)	0.09086 (8)	0.0318 (2)	

### Atomic displacement parameters (Å^2^)

**Table:**

	*U*^11^	*U*^22^	*U*^33^	*U*^12^	*U*^13^	*U*^23^
C1	0.0210 (6)	0.0253 (6)	0.0304 (6)	−0.0036 (4)	0.0142 (5)	−0.0020 (5)
C2	0.0278 (6)	0.0268 (6)	0.0433 (7)	0.0012 (5)	0.0198 (6)	0.0003 (5)
C3	0.0427 (8)	0.0207 (6)	0.0519 (8)	−0.0041 (5)	0.0281 (7)	−0.0042 (5)
C4	0.0341 (7)	0.0272 (7)	0.0473 (8)	−0.0128 (5)	0.0225 (6)	−0.0096 (6)
C5	0.0222 (6)	0.0245 (6)	0.0273 (6)	−0.0033 (4)	0.0139 (5)	−0.0009 (4)
C6	0.0254 (6)	0.0240 (6)	0.0351 (7)	−0.0087 (4)	0.0157 (5)	−0.0029 (5)
C7	0.0247 (6)	0.0305 (7)	0.0362 (7)	−0.0065 (5)	0.0144 (5)	0.0005 (5)
O1	0.0225 (4)	0.0247 (5)	0.0435 (5)	−0.0055 (3)	0.0154 (4)	−0.0040 (4)
O2	0.0270 (5)	0.0219 (5)	0.0486 (6)	−0.0008 (3)	0.0180 (4)	−0.0007 (4)
O3	0.0216 (5)	0.0248 (5)	0.0450 (5)	−0.0056 (3)	0.0160 (4)	−0.0008 (4)

### Geometric parameters (Å, º)

**Table:**

C1—C2	1.3490 (16)	C5—O2	1.2070 (14)
C1—O1	1.3698 (14)	C5—O3	1.3390 (14)
C1—C5	1.4594 (15)	C6—O3	1.4524 (13)
C2—C3	1.4230 (17)	C6—C7	1.5054 (17)
C2—H2	0.95	C6—H6A	0.99
C3—C4	1.3394 (18)	C6—H6B	0.99
C3—H3	0.95	C7—C7^i^	1.528 (2)
C4—O1	1.3622 (14)	C7—H7A	0.99
C4—H4	0.95	C7—H7B	0.99
			
C2—C1—O1	110.40 (10)	O3—C6—C7	107.10 (9)
C2—C1—C5	134.13 (11)	O3—C6—H6A	110.3
O1—C1—C5	115.46 (10)	C7—C6—H6A	110.3
C1—C2—C3	106.27 (11)	O3—C6—H6B	110.3
C1—C2—H2	126.9	C7—C6—H6B	110.3
C3—C2—H2	126.9	H6A—C6—H6B	108.5
C4—C3—C2	106.44 (11)	C6—C7—C7^i^	110.71 (13)
C4—C3—H3	126.8	C6—C7—H7A	109.5
C2—C3—H3	126.8	C7^i^—C7—H7A	109.5
C3—C4—O1	111.02 (11)	C6—C7—H7B	109.5
C3—C4—H4	124.5	C7^i^—C7—H7B	109.5
O1—C4—H4	124.5	H7A—C7—H7B	108.1
O2—C5—O3	124.28 (10)	C4—O1—C1	105.87 (9)
O2—C5—C1	124.83 (10)	C5—O3—C6	115.62 (9)
O3—C5—C1	110.89 (10)		
			
O1—C1—C2—C3	−0.07 (14)	O1—C1—C5—O3	176.33 (9)
C1—C2—C3—C4	0.03 (15)	C1—C5—O3—C6	179.42 (9)
C2—C3—C4—O1	0.02 (15)	C7—C6—O3—C5	178.85 (10)
C3—C4—O1—C1	−0.06 (14)	O3—C6—C7—C7^i^	−178.14 (12)
C2—C1—O1—C4	0.08 (14)	C5—C1—C2—C3	−179.47 (13)
O1—C1—C5—O2	−3.87 (17)	C5—C1—O1—C4	179.61 (10)
C2—C1—C5—O2	175.50 (14)	O2—C5—O3—C6	−0.38 (17)
C2—C1—C5—O3	−4.3 (2)		

Symmetry code: (i) −*x*+3/2, −*y*+1/2, −*z*.

### Hydrogen-bond geometry (Å, º)

*Cg*1 is the centroid of the O1/C1–C4 ring.

**Table:**

*D*—H···*A*	*D*—H	H···*A*	*D*···*A*	*D*—H···*A*
C3—H3···O2^ii^	0.95	2.41	3.3526 (15)	174
C4—H4···O1^iii^	0.95	2.60	3.4142 (18)	145
C4—H4···O2^iii^	0.95	2.49	3.317 (2)	146
C6—H6*B*···*Cg*1^iv^	0.99	2.66	3.5869 (16)	156

Symmetry codes: (ii) *x*, *y*+1, *z*; (iii) −*x*+5/2, *y*+1/2, −*z*+1/2; (iv) −*x*+2, −*y*+1, −*z*.
